# Cardiac involvement in hereditary myopathy with early respiratory failure: A cohort study

**DOI:** 10.1212/WNL.0000000000003064

**Published:** 2016-09-06

**Authors:** Hannah E. Steele, Elizabeth Harris, Rita Barresi, Julie Marsh, Anna Beattie, John P. Bourke, Volker Straub, Patrick F. Chinnery

**Affiliations:** From the John Walton Muscular Dystrophy Research Centre (H.E.S., E.H., R.B., J.M., V.S.), Newcastle University; Department of Cardiology (A.B., J.P.B.), Freeman Hospital, NUTH NHS Foundation Trust; Medical Research Council Mitochondrial Biology Unit (P.F.C.); and Department of Clinical Neurosciences (P.F.C.), School of Clinical Medicine, University of Cambridge, UK.

## Abstract

**Objective:**

To assess whether hereditary myopathy with early respiratory failure (HMERF) due to the c.951434T>C; (p.Cys31712Arg) *TTN* missense mutation also includes a cardiac phenotype.

**Method:**

Clinical cohort study of our HMERF cohort using ECG, 2D echocardiogram, and cross-sectional cardiac imaging with MRI or CT.

**Results:**

We studied 22 participants with the c.951434T>C; (p.Cys31712Arg) *TTN* missense mutation. Three were deceased. Cardiac conduction abnormalities were identified in 7/22 (32%): sustained atrioventricular tachycardia (n = 2), atrial fibrillation (n = 2), nonsustained atrial tachycardia (n = 1), premature supraventricular complexes (n = 1), and unexplained sinus bradycardia (n = 1). In addition, 4/22 (18%) had imaging evidence of otherwise unexplained cardiomyopathy. These findings are supported by histopathologic correlation suggestive of myocardial cytoskeletal remodeling.

**Conclusions:**

Coexisting cardiac and skeletal muscle involvement is not uncommon in patients with HMERF arising due to the c.951434T>C; (p.Cys31712Arg) *TTN* mutation. All patients with pathogenic or putative pathogenic *TTN* mutations should be offered periodic cardiac surveillance.

Hereditary myopathy with early respiratory failure (HMERF) is an autosomal dominant disorder arising due to missense mutations in the fibronectin III domain of the *TTN* gene, most commonly c.951434T>C; (p.Cys31712Arg).^1^ HMERF is characterized by adult onset of distal or proximal muscle weakness in association with early respiratory muscle weakness, which may be the presenting feature and require noninvasive ventilation. Muscle biopsy findings are largely nonspecific, although myofibrillar myopathy and cytoplasmic bodies are described.^2^

Other skeletal myopathies caused by missense mutations in titin include tibial muscular dystrophy due to heterozygous mutations in the C-terminus^3^ and limb-girdle muscular dystrophy type 2J arising from recessive mutations at the same locus.^4^ Cardiac complications in these phenotypes have not been reported previously.^1,3–5^ Conversely, heterozygous truncating *TTN* mutations are a recognized cause of dilated^6^ and restrictive^7^ cardiomyopathies without apparent skeletal muscle involvement. However, the rare coexistence of skeletal and cardiac muscle disease in recessive truncating titin mutations^6^ raises the possibility that cardiac involvement may occur in other titinopathies. This has implications for the surveillance of those at risk. To address this, we carried out the first systematic cardiac study in HMERF using multimodal structural and functional cardiac imaging.

## METHODS

All participants known to the John Walton Muscular Dystrophy Research Centre, Newcastle upon Tyne, United Kingdom, with the c.951434T>C; (p.Cys31712Arg) *TTN* missense mutation had a 12-lead ECG and echocardiogram requested as part of routine clinical care. All available cardiac test results were reviewed. Thereafter, all participants residing within the North East of England were invited to attend for a cardiac MRI, irrespective of cardiac symptomatology or initial findings. Where participants were unable to tolerate MRI, cardiac CT scan was offered.

All cardiac MRIs were performed on a 1.5T Siemens MRI scanner using a standardized cardiomyopathy protocol, with black blood anatomical, multiplanar short tau inversion recovery, multiplanar cines—including short axis stack for ventricular function, multiplanar cines, and delayed enhancement sequences obtained with gadoterate meglumine (Dotarem; Guerbet, Villepinte, France). All cardiac CT imaging was performed on a Siemens dual source CT scanner retrospectively gated at low dose for functional information only with a Flash mode delayed enhancement series 7 minutes following iohexol (Omnipaque; GE Healthcare, Cleveland, OH) administration.

### Histopathologic correlation.

Based on previous reports of desmin as a marker of cardiac dysfunction,^8^ we undertook analysis of frozen myocardial samples collected postmortem from 3 patients with HMERF.

#### Immunohistochemistry.

Immunolabeling for β-spectrin (clone RBC2/3D5), desmin (DAKO M0760; Glostrup, Denmark), myotilin (NCL-Myotilin; Leica Biosystems, Newcastle, UK), VCP (BD Biosciences, East Rutherford, NJ), ubiquitin (NCL-UBIQm; Leica Biosystems), and p62 (Abcam ab56416; Cambridge, UK) was undertaken.

#### Western blot.

Myocardial samples from patients and age-matched controls with no reported cardiac pathology were homogenized and run on sodium dodecyl sulfate polyacrylamide gel electrophoresis (4%–12% gradient). Immunoanalysis was performed using the antibody against desmin. Immunoblots were visualized with SuperSignal West Pico Chemiluminescent Substrate detection using AlphaInnotech FluorChem Q platform and AlphaView software v3.0. All tests were performed in duplicate.

### Standard protocol approvals, registrations, and patient consents.

Clinical assessments were undertaken as routine clinical care. Consent and ethical approval was in place for the histopathologic studies.

## RESULTS

We identified 22 participants with the c.951434T>C; (p.Cys31712Arg) *TTN* mutation. Three were deceased. Eighteen attended for echocardiogram, of whom 6 subsequently had cardiac MRI and 4 cardiac CT imaging. Two individuals failed to attend planned MRI scans. Cross-sectional imaging was not requested in 7 patients due to geographic dispersion. Clinical features are outlined in table 1.

**Table 1:**
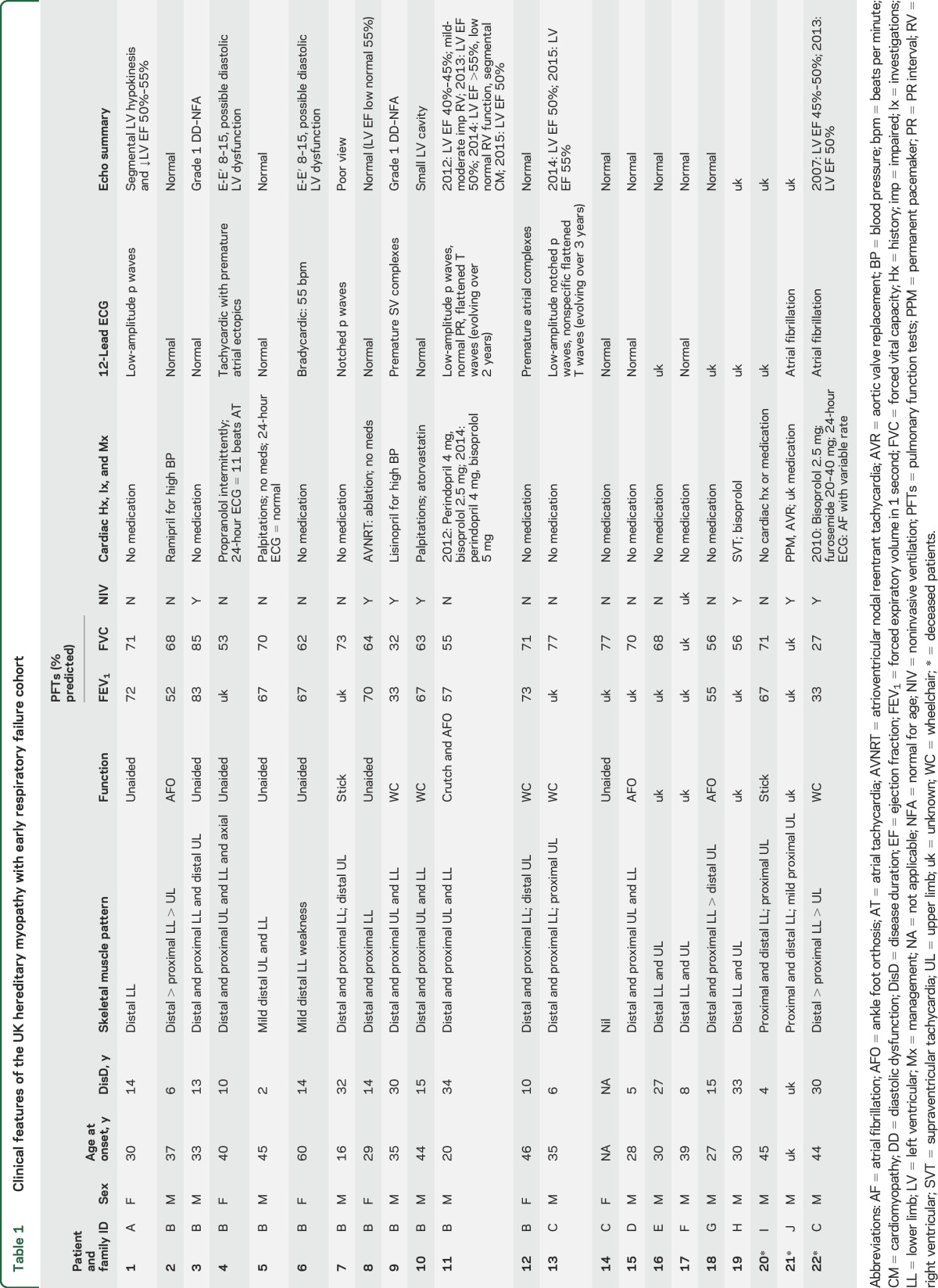
Clinical features of the UK hereditary myopathy with early respiratory failure cohort

We identified conduction abnormalities in 32% (7/22) of patients. These included sustained atrioventricular tachycardia (8B and 19H), nonsustained atrial tachycardia (4B; figure, A), premature supraventricular complexes (9B), unexplained sinus bradycardia (6B), and atrial fibrillation (21J and 22C). Patients 8B and 19H were treated with bisoprolol and 8B underwent catheter ablation. Patients 5B and 10B had a history of palpitations without specific diagnosis being reached despite investigation.

**Figure:**
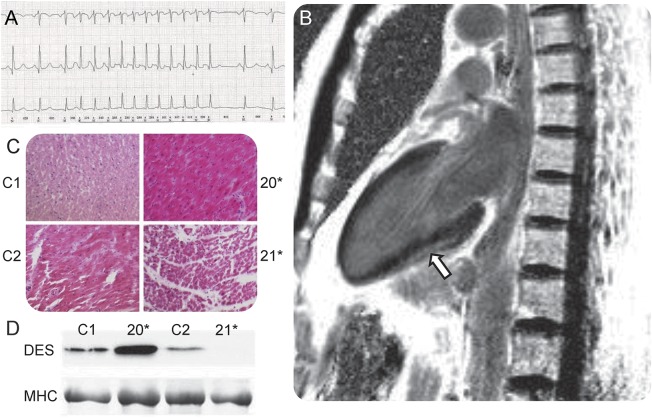
Clinical and pathologic features of cardiac involvement in hereditary myopathy with early respiratory failure (HMERF) (A) Twenty-four-hour ECG demonstrates nonsustained atrial tachycardia (patient 4B). (B) Cardiac MRI of 11B demonstrates subepicardial fibrosis (arrow). (C) Hematoxylin & eosin staining of myocardium in controls (C1 and C2) and patients with HMERF (20* and 21*). (D) Western blot staining for desmin (DES) and myosin heavy chain (MHC).

Asymptomatic global left ventricular systolic dysfunction was evident in 4/22 (18%) patients (1A, 11B, 13C, and 22*) on echocardiogram (tables 1 and 2). Although none had chamber dilation, the findings were compatible with nonischemic cardiomyopathy. Two were known to have reduced left ventricular (LV) ejection fraction at study onset (11B and 13C), and one was identified with mild right ventricular (RV) systolic dysfunction in the course of the study (1A). We identified subepicardial fibrosis in 11B on late gadolinium-enhanced MRI (figure, B). None had other lifestyle, history, or medical risk factors to explain their cardiac features.

**Table 2:**
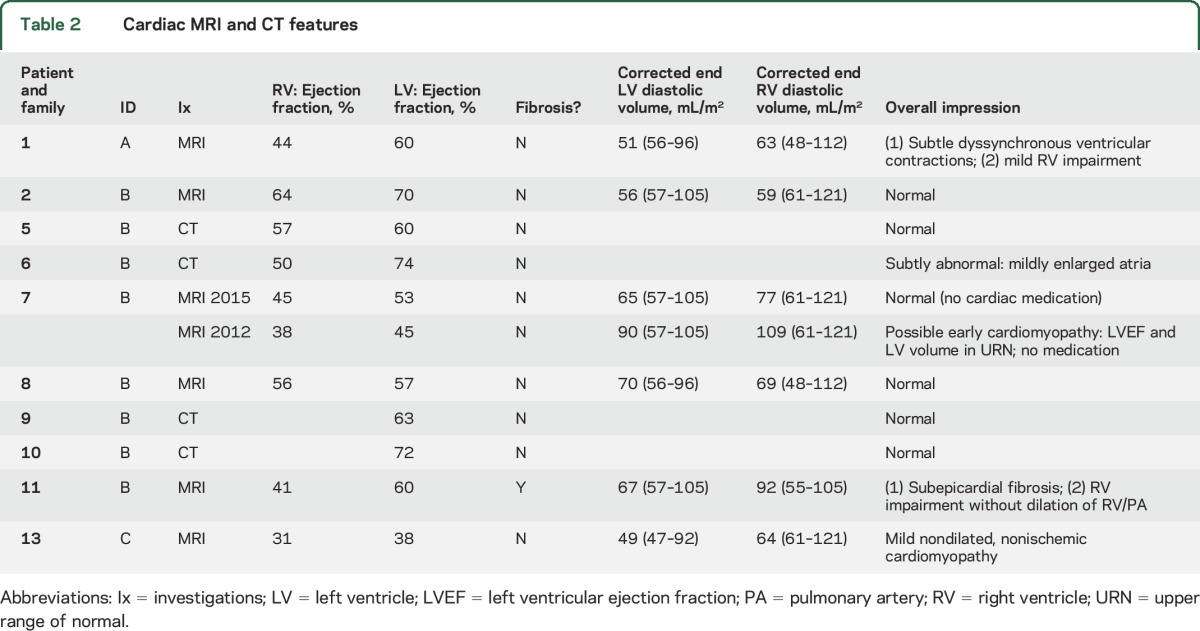
Cardiac MRI and CT features

Four individuals had evidence of possible or definite LV diastolic dysfunction by E-E′ measures on echocardiography. Patient 9B was on maintenance lisinopril for hypertension, but none of the others (3B, 4B, 6B) had any history of cardiac disease, cardioactive medication use, or prior cardiology assessment. Patients 6B and 9B underwent cross-sectional cardiac imaging with CT and mild diastolic impairment was confirmed in 6B (tables 1 and 2).

### Response to treatment.

Participant 11B demonstrated sustained improvement in cardiac function in the 2012–2015 period following initiation of perindopril and bisoprolol therapy. Left ventricular function improved in patient 22* after commencing β-blocker therapy (table 1).

### Relationship to disease onset.

The age range of individuals developing cardiomyopathy overlapped with those without (40–65 and 33–65 years, respectively) and with disease duration (6–34 and 2–33 years, respectively). Cardiomyopathy emerged 4–30 years after first skeletal muscle symptoms (table 1).

### Relationship to respiratory disease.

We assessed the relationship of confirmed ventricular systolic dysfunction at any time with respiratory disease and noninvasive ventilation (NIV) use. No relationship was identified between ventricular systolic impairment and respiratory disease (reduction in pulmonary function tests of 25% or more from predicted) or use of NIV (*p* = 0.2722 and *p* = 0.2778, respectively; Fisher exact test).

### Histopathologic correlation.

#### Immunohistochemistry.

Myocardial tissue preservation was satisfactory in patient 20*, degraded in patient 21* (figure, C), and unsuitable for further analysis in patient 22*. Immunolabeling for myofibrillar proteins was unremarkable and did not demonstrate abnormal protein accumulation (not shown).

#### Immunoblot.

Desmin expression was upregulated (approximately 2.5-fold) in patient 20* compared to controls, suggesting myocardial cytoskeletal remodeling.^8^ Patient 21* showed reduced desmin expression consistent with extensive postmortem delay (figure, D).

## DISCUSSION

Our findings show that cardiac involvement is not uncommon in patients with the c.951434T>C (p.Cys31712Arg) *TTN* missense mutation. Conduction abnormalities occurred in a third of patients, with atrial fibrillation and sustained paroxysmal atrioventricular tachycardia most frequently identified (2/22; 9% each). The prevalence of the latter arrhythmia is significantly higher than seen in the general population (9% vs 0.2%; *p* = 0.0026; Fisher exact test).^9^

Additionally, cardiomyopathy was identified in 18% (4/22). Importantly, this was responsive to standard cardioactive therapies. Interestingly, the presence of either LV or RV dysfunction was independent of respiratory failure, suggesting the mechanism is not secondary to nocturnal hypoventilation, restrictive pulmonary physiology, or cor pulmonale. The etiology of the diastolic dysfunction observed is uncertain given the absence of LV hypertrophy or significant fibrosis. Diastolic dysfunction is a recognized feature of cardiovascular aging and consequently, is the most likely explanation for our findings. However, a disease-specific association cannot be excluded.

As a recently recognized cause of myofibrillar myopathy (MFM), the *TTN* mutation causing HMERF is now included in genetic testing panels for MFM.^2^ Cardiac involvement in other myofibrillar myopathies, also encompassing arrhythmia and cardiomyopathy, is well-recognized, with an estimated prevalence of 30%.^10^ Our findings are in keeping with this.

The main limitation of our study is its pragmatic nature as it was conducted in the context of routine clinical health care. Consequently, the echocardiograms were performed and reported by several—albeit experienced—echo-technicians and the CT scans were reported retrospectively. While MRI remains the gold standard investigation for assessment of ventricular function, use of CT in this population, with neuromuscular respiratory failure and NIV, enabled more patients to undergo cross-sectional cardiac imaging. Although the 2 modalities are not directly comparable, where imaging is undertaken longitudinally using the same method, an assessment of change can be made.

As the full spectrum of cardiac and skeletal muscle phenotypes associated with *TTN* mutations remains unknown, patients with pathogenic or putative pathogenic *TTN* mutations should be offered periodic cardiac surveillance. However, based on the findings we present here, some of the observed abnormalities may be due to normal aging, and not *TTN* cardiomyopathy per se.

## GLOSSARY

HMERF: hereditary myopathy with early respiratory failure
LV: left ventricular
MFM: myofibrillar myopathy
NIV: noninvasive ventilation
RV: right ventricular

## References

[1] Pfeffer G, Elliott HR, Griffin H, et al. Titin mutation segregates with hereditary myopathy with early respiratory failure. Brain 2012;135:1695–1713. 22577215 10.1093/brain/aws102 PMC3359754

[2] Pfeffer G, Barresi R, Wilson IJ, et al. Titin founder mutation is a common cause of myofibrillar myopathy with early respiratory failure. J Neurol Neurosurg Psychiatry 2014;85:331–338. 23486992 10.1136/jnnp-2012-304728 PMC6558248

[3] Hackman P, Vihola A, Haravuori H, et al. Tibial muscular dystrophy is a titinopathy caused by mutations in TTN, the gene encoding the giant skeletal-muscle protein titin. Am J Hum Genet 2002;71:492–500. 12145747 10.1086/342380 PMC379188

[4] Haravuori H, Makela-Bengs P, Udd B, et al. Assignment of the tibial muscular dystrophy locus to chromosome 2q31. Am J Hum Genet 1998;62:620–626. 9497249 10.1086/301752 PMC1376946

[5] Udd B, Partanen J, Halonen P, et al. Tibial muscular dystrophy: late adult-onset distal myopathy in 66 Finnish patients. Arch Neurol 1993;50:604–608. 8503797 10.1001/archneur.1993.00540060044015

[6] Herman DS, Lam L, Taylor MR, et al. Truncations of titin causing dilated cardiomyopathy. N Engl J Med 2012;366:619–628. 22335739 10.1056/NEJMoa1110186 PMC3660031

[7] Peled Y, Gramlich M, Yoskovitz G, et al. Titin mutation in familial restrictive cardiomyopathy. Int J Cardiol 2014;171:24–30. 24315344 10.1016/j.ijcard.2013.11.037

[8] Monreal G, Nicholson LM, Han B, et al. Cytoskeletal remodeling of desmin is a more accurate measure of cardiac dysfunction than fibrosis or myocyte hypertrophy. Life Sci 2008;83:786–794. 18955067 10.1016/j.lfs.2008.09.026

[9] Orejarena LA, Vidaillet H, DeStefano F, et al. Paroxysmal supraventricular tachycardia in the general population. J Am Coll Cardiol 1998;31:150–157. 9426034 10.1016/s0735-1097(97)00422-1

[10] Semmler AL, Sacconi S, Bach JE, et al. Unusual multisystemic involvement and a novel BAG3 mutation revealed by NGS screening in a large cohort of myofibrillar myopathies. Orphanet J Rare Dis 2014;9:121. 25208129 10.1186/s13023-014-0121-9 PMC4347565

